# Brain nuclear receptors and cardiovascular function

**DOI:** 10.1186/s13578-023-00962-3

**Published:** 2023-01-20

**Authors:** Mengjie Wang, Yongjie Yang, Yong Xu

**Affiliations:** 1 Department of Pediatrics, USDA/ARS Children’s Nutrition Research Center, Baylor College of Medicine, Houston, TX USA; 2 Department of Molecular and Cellular Biology, Baylor College of Medicine, Houston, TX USA

**Keywords:** Nuclear receptor, Central nervous system, Cardiovascular function, Brain–heart interaction

## Abstract

Brain–heart interaction has raised up increasing attentions. Nuclear receptors (NRs) are abundantly expressed in the brain, and emerging evidence indicates that a number of these brain NRs regulate multiple aspects of cardiovascular diseases (CVDs), including hypertension, heart failure, atherosclerosis, etc. In this review, we will elaborate recent findings that have established the physiological relevance of brain NRs in the context of cardiovascular function. In addition, we will discuss the currently available evidence regarding the distinct neuronal populations that respond to brain NRs in the cardiovascular control. These findings suggest connections between cardiac control and brain dynamics through NR signaling, which may lead to novel tools for the treatment of pathological changes in the CVDs.

## Introduction

Nuclear receptors (NRs) contain a large superfamily of at least 48 DNA-binding transcription factors whose target genes are critical for many biological processes, including growth, differentiation, development, and metabolism [1, 2]. The NR superfamily, sharing common structure, can be divided into three subfamilies: (1) steroid hormone receptors, such as estrogen receptor (ER), androgen receptor (AR), glucocorticoid receptor (GR), mineralocorticoid receptor (MR), and progesterone receptor (PR); (2) non-steroid hormone receptors, such as thyroid hormone receptor (THR), retinoic acid receptor (RAR), and vitamin D receptor (VDR); (3) orphan receptors, such as testicular nuclear receptor (TR), retinoid X receptor (RXR), peroxisome proliferator-activated receptor (PPAR), liver X receptor (LXR), steroidogenic factor 1 (SF-1) [3].

NRs sense changing levels of nutrients and hormones and it has been well established that NRs play essential roles in the regulation of various diseases including dyslipidemia, diabetes, cancer, neurodegenerative disorders, infertility, and obesity [4]. More recently, the interaction of NRs and cardiovascular function has received increasing attentions [5, 6]. In this review, we will focus on the relationship between brain NRs and cardiovascular diseases (CVDs). NRs are found abundantly expressed in the brain [7–10]. Emerging evidence indicates that a number of these brain NRs regulate multiple aspects of CVDs, including hypertension, atherosclerosis, and heart failure [11–14]. Here, we classified the NRs as three groups: (1) NRs (ER, MR, and THR) that have direct evidence supports the brain NRs regulate cardiovascular functions; (2) NRs (AR, GR, PPAR, PR, RAR, RXR, VDR, etc.) that play critical roles in the regulation of cardiovascular functions but lack direct evidence to establish the relationship between brain NRs and cardiovascular functions; (3) NRs (LXR, SF-1, TR, etc.) that currently have no known roles in the regulation of cardiovascular or other functions.

An imbalanced brain–heart interaction has a negative impact on cardiovascular health [15, 16]. In this review, we summarize all the current and available evidence regarding the role of brain NRs in the regulation of cardiovascular function.

## Brain NRs that regulate cardiovascular functions

### Estrogen receptor (ER)

Estrogen exerts a vast range of biological effects in cardiovascular function, including vascular function, the inflammatory response, metabolism, insulin sensitivity, cardiac myocyte and stem cell survival, and the development of hypertrophy [17]. Estrogen, with its potential as a cardioprotective agent and as an immunomodulator of the inflammatory response in atherosclerosis, has been widely discussed during the past three decades [18–20]. CVDs are the leading cause of death among women [21]. Women of reproductive age have lower blood pressure than men and can be protected from atherosclerosis [22]; while the loss of endogenous estrogen production after menopause leads to a higher prevalence of hypertension and incidence of stroke than men [23]. While in postmenopausal women [24] and ovariectomized (OVX) rats [25], estrogen replacement decreases blood pressure. The studies [26, 27], investigating the sex differences in CVDs, emphasize the importance and urgent need to further understand of estrogen and estrogen receptors (ER) in cardiovascular function.

Estrogen signaling is mediated through two ERs, ERα and ERβ, both belonging to the NR family of transcription factors. Recent studies have demonstrated that activated ER may promote physiological functions via non-transcriptional mechanisms [12, 28]. These non-genomic effects involve steroid-induced modulation of cytoplasmic or cell membrane-bound regulatory proteins. Several biological actions of estrogen are associated with these non-genomic signaling, and intracellular regulatory cascades. For example, extracellular signal-regulated kinase/mitogen-activated protein kinases (ERK/MAPK) and tyrosine kinases or the modulation of G-protein-coupled receptors have been shown to be non-transcriptionally recruited by estrogen in diverse tissues. In the heart and vasculature, the nongenomic mechanisms underlie estrogen induced short-term arterial vasodilation, inhibition of atherosclerotic lesions, and amelioration of ischemia/reperfusion-induced cardiac injury [12, 28].

ER mRNA and protein have been detected in both peripheral tissues and central nervous system [7–9]. ERα and ERβ immunoreactivity (ir) were found primarily localized to cell nuclei within select regions of the brain, including the olfactory bulb, cerebral cortex, septum, preoptic area, bed nucleus of the stria terminalis, amygdala, paraventricular hypothalamic nucleus, thalamus, ventral tegmental area, substantia nigra, dorsal raphe, locus coeruleus, and cerebellum. Extranuclear-ir was detected in several areas, including fibers of the olfactory bulb, CA3 stratum lucidum, and CA1 stratum radiatum of the hippocampus and cerebellum. In detail, nuclear ERα-ir was the predominant subtype in the hippocampus, preoptic area, and most of the hypothalamus, whereas it was sparse or absent from the cerebral cortex and cerebellum [7–9]. ERα and ERβ have distinct tissue expression patterns in both humans and rodents [7, 8, 29, 30], and gene-targeted animal models lacking these receptors exhibit distinct phenotypes [9, 31].

**Fig. 1:**
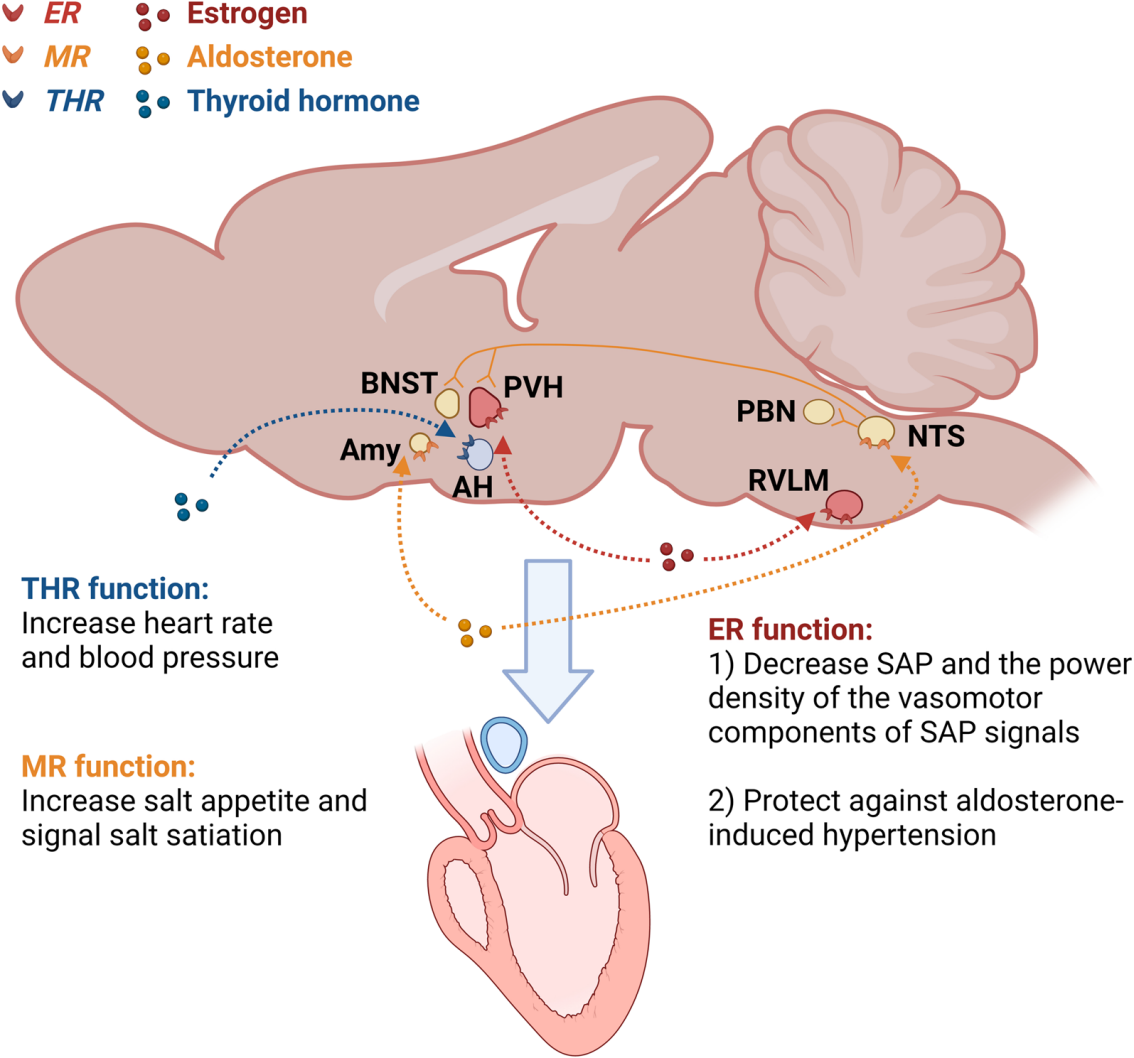
Schematic of the brain nuclear receptors regulate cardiovascular function. ER in the RVLM and the PVH decreases SAP and protects against aldosterone-induced hypertension; THR in the AH increases heart rate and blood pressure; MR in the NTS and the Amy increases salt appetite and signals salt satiation. MR-NTS neurons project to the PBN, PVH, and BNST, modulating sensory coding of salt taste and signaling salt satiation. *ER* estrogen receptor, *RVLM* rostral ventrolateral medulla, *PVH* paraventricular nucleus of the hypothalamus, *SAP* systolic arterial pressure, *THR* thyroid hormone receptor, *AH* anterior hypothalamus, *MR* mineralocorticoid receptor, *NTS* nucleus tractus solitarius, *Amy* amygdala, *PBN* parabrachial nucleus, *BNST* bed nucleus of the stria terminalis

The rostral ventrolateral medulla (RVLM), where sympathetic premotor neurons are located, is a key region involved in regulation of blood pressure and hypertension [32]. ERα and ERβ are expressed in the RVLM [12, 13]. In rats, bilateral microinjection of 17β-estradiol in the RVLM elicited short-term cardiovascular depressive effects, while this effect was abolished by co-injection of ER antagonist [12]. This study further identified that ERβ, instead of ERα, induced significant decrease in systemic arterial pressure (SAP) and the power density of the vasomotor components of SAP signals [12]. Several studies have revealed the potential mechanisms in the 17β-estradiol-induced cardiovascular depressive responses, including the calcium current inhibition decrease in sympathetic tone [33] and the engagement of the inducible nitric oxide synthase (iNOS)-derived NO in the RVLM [12]. In addition to ERβ, extranuclear ERα also indirectly modulate the function of RVLM C1 bulbospinal neurons in selected afferents [33]. The high level of oxidative stress in the RVLM contributes to increased sympathetic outflow and hypertension [34], which is frequently seen in postmenopausal women. These studies emphasize that ERs in the RVLM is critical in the regulation of cardiovascular function (Fig. 1).

The paraventricular nucleus of the hypothalamus (PVH) is another important site in the regulation of cardiovascular function [13, 35]. PVH neurons projecting to the RVLM play a regulatory role in the determination of the sympathetic outflow [35]. Using selective ER agonists and recombinant adeno-associated virus (AAV) carrying small interference (si) RNA to silence either ERα (AAV-siRNA-ERα) or ERβ (AAV-siRNA-ERβ) in the PVH and RVLM, researchers found that both PVH and RVLM ERβ, instead of ERα in these nuclei, contribute to the protective effects of estrogen against aldosterone-induced hypertension [13].

Mean arterial pressure and renal sympathetic nerve activities are significantly decreased following injection of estrogen into various brain regions including the nucleus tractus solitarius (NTS), RVLM, parabrachial nucleus (PBN), central nucleus of the amygdala (CNA) and the intrathecal space in estrogen-replaced rats [36]. Heart rate and vagal parasympathetic nerve activities are significantly decreased following injection of estrogen into the NTS, nucleus ambiguus (Amb), PBN and the intrathecal space [36]. This study underscores the importance of ER in other brain regions in the regulation of cardiovascular function. Further studies including using genetic rodent models are needed to further understand the regulatory role of ER in different brain regions in the regulation of cardiovascular function.

ERα is believed to be the primary ER that mediates estrogenic actions to prevent obesity and diabetes [37, 38], contributors to the prevalence of CVDs. The various brain regions responsible ERα’s regulatory effects on body weight and glucose homeostasis have been well studied. For example, ERα in amygdala neurons regulates body weight and ERα-expressing neurons in the ventrolateral subdivision of the ventromedial hypothalamic nucleus regulate glucose homeostasis [39–41]. However, the brain regions responsible for the direct and protective effects of ERα in cardiovascular function still need to be determined.

### Mineralocorticoid receptor (MR)

Mineralocorticoids are a class of corticosteroids produced in the adrenal cortex, which influence salt and water balances (electrolyte balance and fluid balance). The primary mineralocorticoid is aldosterone [42, 43]. Increased levels of aldosterone tend to cause myocardial hypertrophy and remodeling, fibrosis, apoptosis, endothelial dysfunction, and impaired myocardial perfusion, which increasing the incidence of cardiovascular events [44].

The mineralocorticoid receptor (MR), also known as the aldosterone receptor, is a protein that in humans is encoded by the NR3C2 gene [45]. Aldosterone binding to MR results in reabsorption of sodium and water in exchange for potassium in various sites, causing an increased intravascular fluid retention and volume overload. This undesired impact in fluid balance further causes hypervolemia and promotes congestion in patients with chronic heart failure [42].

In addition, MR activation promotes hypertension, fibrosis, and inflammation [45, 46], while pharmacological blockade of the MR has protective effects in animal models of CVDs [47, 48] and preventive effects against mortality and morbidity in heart failure patients [49, 50]. Thus, a better understanding of the implications of the MR in the setting of CVDs is critical for refining treatments and improving patient care.

MR is considered as a ubiquitous transcription factor, which is expressed in peripheral tissues such as heart, kidney, salivary and sweat glands, skin, and also in central nervous system including hippocampus and hypothalamus [51]. In 1968, Bruce McEwen discovered that ^3^H-corticosterone administered to adrenalectomised rats is retained in neurons of hippocampus [52]. Since its discovery, a great body of studies were conducted to study the role of brain MR function in the cardiovascular regulation [53–55], especially the contributory role of salt intake [56, 57]. The NTS was found to be richly endowed with MR and the role of MR-NTS neurons in salt appetite regulation has been widely studied [58–60]. Neurons that abundantly express MR are located in the dorsal and ventral hippocampal CA1-4 pyramidal cell fields and the dentate gyrus, amygdala and lateral septum [53–55]. The MR-NTS neurons connect to the nearby area postrema, a periventricular region that is readily accessible to peptides and other compounds in the 4th ventricle. Intracerebroventricular (i.c.v.) infusion of the MR agonist aldosterone in the 4th ventricle increased salt appetite [59], while MR knockdown in the NTS prevented this aldosterone-induced salt appetite [60]. MR-NTS neurons project to the parabrachial nucleus, amygdala, paraventricular nucleus, hippocampus, and the bed nucleus of the striae terminalis (BNST), which subsequently signal salt satiation [58, 61, 62]. This neuronal network explained why interventions in the amygdala affected aldosterone regulation of salt appetite [63]. These studies highlight the importance of MR-NTS neurons in the regulation of cardiovascular function via regulation of salt appetite (Fig. 1).

### Thyroid hormone receptor (THR)

Thyroid hormone (TH) has profound effects on cardiovascular function [64, 65]. TH increases myocardial inotropy and heart rate and dilates peripheral arteries to increase cardiac output (CO). Triiodothyronine (T_3_), the active form of TH, binds to NR proteins and mediates effects on the systemic vasculature include relaxation of vascular smooth muscle, resulting in decreased arterial resistance and diastolic blood pressure [65]. In patients with hyperthyroidism, cardiac contractility and cardiac output are enhanced and systemic vascular resistance is decreased, while in patients with hypothyroidism, the opposite phenotype is seen [66, 67]. In addition, the phenotype of the failing heart resembles that of the hypothyroid heart, both in cardiac physiology and in gene expression [68]. Changes in serum T_3_ levels in patients with chronic congestive heart failure are caused by alterations in TH metabolism suggesting that patients may benefit from T_3_ replacement in this setting [69]. The literature outlining the effect of TH replacement therapy for reducing the risk of CVDs have been comprehensively reviewed [66, 70–73]. Animal models showed that T_3_ deficiency in the brain influenced all aspects of brain development and physiology [74, 75], which urges the need of a complete understanding of T_3_ action in the brain.

TH receptors (THRs) have isoforms of THRs, THRα1, THRβ1 and THRβ2, which are predominantly responsible for mediating TH action [76, 77]. THRs are encoded by two genes called *Thra* and *Thrb* in mice, or *THRA* and *THRB* in humans [78]. THRs are transcription factors, which act mainly as heterodimers with RXRs. THR/RXR heterodimers bind to DNA at T_3_ response elements and regulate transcription initiation of target genes [78]. THRα1 mRNA is ubiquitous in the rodent brain [79], while THRβ1 mRNA only appears in the brain at a late developmental stage and in few cell types, predominantly in the hypothalamus, pituitary, retina, and cochlea [80]. Accordingly, most functional studies conclude that there is a predominant role of THRα1 in brain development, and a selective influence of THRβ1/2 in the development of sensory functions and the maturation of some specific neuronal populations [81].

In mice, THRα1 mRNA is predominantly expressed in the heart and brain, whereas THRβ1 is expressed in peripheral tissue including skeletal muscle, kidney, and liver [82]. THRα1 mRNA was widely expressed in the rat fetal neocortical plate, site of cortical neuronal differentiation, while THRβ1 transcripts were restricted in distribution, with prominent expression in zones of neuroblast proliferation such as the germinal trigone and the cortical ventricular layer [83].

The cardiovascular and metabolic effects of TH were previously thought to be mediated predominantly by THR isoforms expressed by peripheral tissues, including heart, skeletal muscle, and fat [84, 85]. However, recent studies suggest that TH can also modulate developmental, metabolic, and cardiovascular processes by acting on specific targets in the brain [86–88]. Mice expressing the mutant THRα1R384C displayed a mild bradycardia, accompanied by a reduced expression of nucleotide-gated potassium channel 2 mRNA in the heart [88]. Another study identified a previously unknown population of parvalbuminergic neurons in the anterior hypothalamus (AH) that requires THR signaling for proper cardiovascular function [14]. In this study, specific stereotaxic ablation of these cells in the mouse resulted in hypertension and temperature-dependent tachycardia [14], indicating a role in the central autonomic control of blood pressure and heart rate (Fig. 1). More studies are needed to address the role of brain THRs in the regulation of cardiovascular function.

## NRs that regulate cardiovascular functions with unclear brain connection

### Estrogen-related receptor (ERR)

Estrogen-related receptor (ERR), sharing sequence homology with ER, belongs to the subfamily of orphan NRs. ERRs have three family members, ERRα, ERRβ, and ERRγ [89–91]. ERRα has long been thought to not require an endogenous ligand, because it is activated by the transcriptional coactivator peroxisome proliferator-activated receptor γ (PPARγ) coactivator 1α (PGC-1α), which is necessary for postnatal cardiac mitochondrial biogenesis [92–94]. ERRα serves as a critical nodal point in the regulatory circuitry downstream of PGC-1α to direct the transcription of genes involved in mitochondrial energy-producing pathways in cardiac muscle [95]. One recent study showed that cholesterol is a potential endogenous ligand for ERRα that increased ERRα transcriptional activity [96]. This finding explained why ERRα is constitutively active because cholesterol is ubiquitous. Animal studies showed that knockdown the expression of ERRα and γ in heart after birth caused cardiomyopathy with an arrest in mitochondrial maturation [97], and ERRα null mice subjected to left ventricular pressure overload developed heart failure including chamber dilatation and reduced left ventricular fractional shortening [98]. In addition, using siRNA or inverse agonists to inhibit ERRα function impairs induction of mitochondrial gene expression and respiration by PGC-1α, which predisposes to cardiac remodeling [99, 100]. Disruption of ERRγ gene in mice blocked the high levels of expression in the fetal and postnatal mouse heart and resulted in lactatemia, electrocardiographic abnormalities, and death during the first week of life [101]. These studies indicate that ERRα and ERRγ are necessary for the maturation of both mitochondrial and structural processes during postnatal cardiac development and the adaptive bioenergetic response to hemodynamic stressors known to cause heart failure. Notably, ERRβ transcripts were increased in the fetal ERRα/γ knockdown mouse model, suggesting possibly as a compensatory regulatory response [97]. In addition, ERRβ has distinct functions in regulating energy metabolism in the adult cardiomyocyte, as shown that ERRβ is involved in maintaining maximal ATP generation in contracting cardiomyocytes [102]. ERRs have emerged as potential therapeutic targets for the treatment of heart failure in humans [103].

ERRα and ERRγ are both widely expressed in the brain [104, 105], while ERRβ expression is restricted to the hindbrain [106]. In the mouse brain, ERRα is abundantly expressed in several cortical regions, including orbitofrontal cortex, medial prefrontal cortex (mPFC), and cingulate cortex; highly expressed in the primary and secondary somatosensory cortex, piriform cortex, retrosplenial granular cortex, hippocampus, thalamus, mammillary nucleus, cerebellum, and several brainstem nuclei involved in transmission of sensory information; moderate levels of expression are observed in dorsolateral aspects of the striatum, septum, global pallidus, lateral hypothalamic area, and some midbrain structures including tegmentum and substantia nigra [104]. ERRγ transcripts were abundantly present in the isocortex, the olfactory system, cranial nerve nuclei and major parts of the coordination centers, e.g. reticular formation and major parts of the extrapyramidal motor systems. In addition, ERRγ expression was detected in trigeminal ganglion neurons [105]. There are a lot of studies investigating the functions of ERs and ERRs in the central regulation of energy homeostasis, learning and memory, and social behaviors [104], but the cardiovascular function is still largely unknown.

### Androgen receptor (AR)

Low testosterone levels and androgen deprivation therapy are associated with increased atherosclerosis burden and increased risk of CVDs [107–109]. In men with heart failure, the testosterone replacement therapy is a significant predictor for an increase in peak oxygen consumption on exercise testing [110]. Acute and chronic administration of testosterone both demonstrate beneficial effects in the cardiovascular function [111, 112]. Intravenous administration of testosterone acutely increased cardiac output and reduced peripheral vascular resistance [111]. While during chronic testosterone replacement therapy, circulating levels of inflammatory mediators were reduced, potentially leading to a reduction of left ventricular muscle fibrosis [112].

The androgen receptor (AR) is activated by binding any of the androgenic hormones, including testosterone and dihydrotestosterone [113]. In male mice, the androgen-AR system regulates normal cardiac growth, cardiac adaptive hypertrophy and fibrosis during the process of cardiac remodeling under hypertrophic stress [113, 114]. Global knockout of AR in mice results in reduced heart size, impaired contraction, exacerbation of angiotensin II-induced cardiac fibrosis [114]. Depletion of ARs on the apolipoprotein E-deficient background increases atherosclerosis burden in male mice [115].

Besides the prostate, ARs are expressed in several other tissues including the brain [10, 116]. AR neurons are basically localized in limbic areas such as the hypothalamus [10]. In the rodent, brain AR distribution was distributed in the bed nucleus of the stria terminalis, lateral septum, medial amygdala, medial preoptic area, and ventromedial hypothalamic nucleus [117]. The neuron-AR knockout mice generated using the Nestin-Cre showed that central AR is required in testosterone-induced regulation of male-typical behaviors and gonadotrope and somatotropic axes [118]. While using the Synapsin-Cre, another group reported that neuronal AR deficiency leads to reduced insulin sensitivity in middle-aged mice [119]. Female neuron-AR knockout mice showed impaired kisspeptin/GnRH/LH cascade leading to compromised ovarian follicle dynamics [120]. However, whether the neuron-AR also regulates cardiovascular function is unknown yet.

### Glucocorticoid receptor (GR)

Glucocorticoids are another class of corticosteroids, which are under regulation by the hypothalamic–pituitary–adrenal gland axis [121]. Research in both humans and animals have unraveled that glucocorticoid and their actions via binding to glucocorticoid receptor (GR) are highly involved in the genesis and development of CVDs [122–124]. Synthetic glucocorticoids are commonly prescribed in diverse CVDs, including infectious conditions such as rheumatic fever and myocarditis, structural conditions such as conduction defects and cardiomyopathy, and vascular conditions such as angina and acute myocardial infarction [125]. However, the severe side effects in diabetes, abdominal obesity, and hypertension, all of which are risk factors for CVDs, limit the therapeutic benefits of (synthetic) glucocorticoids [125]. Thus, it is essential to have comprehensive insight into the specific functions of glucocorticoids in the cardiovascular system.

The GR is activated either by the endogenous steroid hormone cortisol or by exogenous glucocorticoids and acts within the cardiovascular system via both genomic and non-genomic pathways. Polymorphisms of the GR are also reported to influence the progress and prognosis of CVDs [126]. The GR exerts numerous functions in the whole body including the brain [127–129]. In the brain, it has been implicated in feedback regulation of the hypothalamic–pituitary–adrenal axis, with potential deficits during aging and in depression. GRs are abundantly expressed in the hippocampus of rodent and human, indicating that this region can form an important target for corticosteroid effects [127]. The NTS abundantly expresses the GR and is a key brain region for processing autonomic and endocrine stress responses mediated by the HPA axis [128, 129]. One recent study found that ablated of GR in the hindbrain of mouse resulted in stress-related behavior and elevated circulating corticosterone and severe adrenal cortex disruption [130]. However, the direct role of brain GR in the regulation of cardiovascular function is still unclear.

### Peroxisome proliferator-activated receptor (PPAR)

Peroxisomes are unique subcellular organelles that sequester diverse oxidative reactions [131]. Peroxisome proliferator-activated receptors (PPARs) are transcription factors of NR superfamily which also include RAR, ER, TR, GR, vitamin D receptor, and several other proteins involved in xenobiotic metabolism. PPARs act on DNA response elements as heterodimers with the retinoid X receptor (RXR). The PPARs, comprising of three subtypes: PPARα, PPARγ, and PPARβ/δ, have overlapping as well as different expression patterns and distinct functions in the metabolic control [4, 132, 133]. Besides expression in various peripheral tissues including liver, heart, kidney, adipose tissue, skeletal muscle and [132], PPAR mRNA and protein distribution in the brain were also investigated [134–138]. The expression of PPAR mRNA and protein in coarse brain regions as well as caudate putamen, hippocampus, hypothalamus, and thalamus were identified in rats [134, 135]. One recent study in mice showed that PPARα, PPARγ, and PPARβ/δ mRNA and protein were found to express in prefrontal cortex, nucleus accumbens, amygdala and ventral tegmental area [136]. Among the isotypes, PPARβ/δ was most widely and highly expressed across brain regions [137, 138] and PPARα was more highly expressed than PPARγ in all brain regions except the prefrontal cortex [136].

Lipid-derived substrates are their natural activating ligands. Omega-3 fatty acids are one kind of the natural ligands of PPARα [139], and their beneficial effects in cardiovascular functions are considered to be conveyed via PPARα [140]. Omega-3 fatty acids are also used to treat hyperlipidemia and hypertension and can significantly reduce the risk for sudden death caused by cardiac arrhythmias and all-cause mortality in patients with known coronary heart disease [141]. Like PPARα, PPARβ/δ improves cardiac function and ameliorates the pathological progression of cardiac hypertrophy, heart failure, cardiac oxidative damage, ischemia–reperfusion injury, lipotoxic cardiac dysfunction and lipid-induced cardiac inflammation [142, 143]. Furthermore, PPARβ/δ activation protects against diabetic cardiomyopathy [144, 145]. Previous studies showed that PPARβ/δ expression was decreased in cardiac muscle during the hyperglycemic state in diabetes mellitus, while in contrast, overexpression of this PPARβ/δ in cardiac cells diminished lipid accumulation and increased glucose metabolism [144, 145]. During the past decade, a large body of studies have demonstrated that PPARγ plays an important role in the cardiovascular system [146, 147]. Thiazolidinediones (TZDs) such as rosiglitazone and pioglitazone, are ligands for PPARγ, have long been used to treat type 2 diabetes and serve as a therapeutic target for CVDs [148]. Besides, PPARγ activation has a role against inflammation and suppresses the inducible nitric oxide synthase (iNOS) upregulation and reactive oxygen species (ROS) production [149].

The mRNA expression of PPARγ in hypothalamus is several folds higher than PPARα and PPARβ/δ [150]. PPARγ immunoreactivity was primarily observed in several brain regions including ARH, PVH, hypothalamic ventromedial neurons (VMH), the lateral hypothalamic area, and tyrosine hydroxylase-containing neurons in the ventral tegmental area [151]. Mice with neuron specific PPARγ knockout (PPARγ BKO) had reduced food intake and weight gain during high fat diet (HFD) feeding. When treated with rosiglitazone, these mice were resistant to rosiglitazone-induced hyperphagia and weight gain [152]. These findings suggest the critical effects of brain PPARγ on food intake, energy expenditure, and insulin sensitivity. However, the question of whether brain PPARs directly regulates cardiovascular function is still unanswered.

### Progesterone receptor (PR)

Accumulated evidence has shown that progesterone exhibits beneficial effects on the cardiovascular function such as lowers blood pressure, inhibits coronary hyperactivity and has powerful vasodilatory and natriuretic effects [153]. Progesterone receptors (PRs), like other sex steroid hormone receptors, are members of a superfamily of ligand-activated transcription factors that regulate gene expression following hormone binding [154]. PRs are expressed in vascular cells [155, 156], and PR expression in vascular tissues is induced by estrogen [156]. One recent study found that ovariectomized female PR knockout mice had significantly greater vascular medial hypertrophy and vascular smooth muscle cell proliferation in response to vascular injury than did wildtype mice [154]. These findings indicate that an important role for the PR in regulating the response to vascular injury and vascular smooth muscle cell proliferation. Novel evidence showed that membrane PRs (mPRs), in addition to nuclear PRs, are also candidates for the rapid and cell-surface initiated progesterone actions in endothelial and smooth muscle vascular cells. Three mPR gene isoforms mPRα (PAQR VII), mPRβ (PAQR VIII), and mPRγ (PAQR V) identified in humans [157], are putative G protein-coupled receptors (GPCRs) for progesterone [158]. mPRβ human mRNA was found in the brain including spinal cord, cerebral cortex, cerebellum, pituitary, thalamus, and caudate nucleus [157], while mPRα and mPRβ rodent mRNA were seen hypothalamus [159, 160]. Several studies have examined the role of hypothalamic PR in the regulation of reproductive functions [161–163]. Microinjection of the PR antagonist, RU486, into the hypothalamic arcuate nucleus shortened LH pulse interval in the progesterone treated rats, while into the hypothalamic anteroventral periventricular nucleus reversed the prolonged cycle length and rescued the progesterone blockade LH surge [161]. Knockout of PR in kisspeptin neurons (enriched hypothalamic anteroventral periventricular and arcuate nuclei) in mice causes loss of LH surges, irregular estrous cycle, and infertility [162, 163]. Compared to the extensive studies regarding PR in reproductive function, the brain PRs in cardiovascular regulation are still largely unknown.

### Retinoid acid receptor (RAR)

Retinoic acid (RA), the derivatives including all-trans RA, 13-cis RA, or 9-cis RA, carries out essential roles in heart development and after birth in the heart’s remodeling response to injury and disease [164, 165]. During early stages of cardiogenesis, alterations in RA levels are often associated with congenital heart defects such as abnormal folding and septation of the outflow tract and cardiac chambers [166]. Furthermore, RA increases myocyte cross-sectional area and induces cardiac hypertrophy in a dose-dependent way [167]. All-trans RA exerts its actions mainly through binding to RA receptor (RAR), while its enantiomer 9-cis RA binds to RAR or retinoid X receptor (RXR) [168].

RAR and RXR each has three different isoforms, termed α, β, and γ, which have overlapping as well as distinct expression patterns and functions during embryogenesis [169]. In vitro study showed that activation of RAR/RXR signaling protects cardiomyocytes from hyperglycemia, by reducing oxidative stress and inhibition of the renin-angiotensin system [170]. In addition, the selective RARβ_2_ agonist AC261066 reduced oxidative stress in an ex vivo murine model of ischemia/reperfusion [171] and later the same group performed in vivo study to show that RARβ_2_ agonist attenuated the development of contractile dysfunction and maladaptive remodeling in post-ischemic heart failure [172]. These studies identified that RARβ_2_ agonist as a potential target in the treatment of heart failure. However, there is lack of direct evidence to establish the relationship between brain RAR and cardiovascular regulation. 

### Retinoid X receptor (RXR)

The three RXR isoforms show tissue-specific expression, while their functions are partially overlapped. RXRα is predominantly expressed in metabolically active systems, such as liver, muscle, lung, kidney, and heart; RXRβ is ubiquitously expressed; whereas RXRγ is abundantly expressed in brain and skeletal muscle [173, 174].

RXR can form heterodimers with many other NRs including the RAR, THR, VDR, liver X receptor (LXR), PPARs, farnesoid X receptor, pregnane X receptor, and CAR [175]. RAR/RXR form heterodimers and regulate RA-induced expression of genes that control cell fate specification, proliferation, and differentiation [176]. RAR/RXR knockout mice developed cardiovascular dysfunction including heart malformations, defects in the conduction system and heart failure [177]. Overexpression of RAR or RXR induces dilated cardiomyopathy, depressed cardiac function, and congestive heart failure [178]. Due to the complex dimerization pattern, it is difficult to conclude whether a particular defect in RXR null mice is attributed to impaired RAR/RXR signaling or to an impairment is signaling by other heterodimeric partners of RXR or by RXR homodimers [176].

RXR mediates brain clean-up after stroke [179]. Deleted RXRα in myeloid phagocytes demonstrated worsened late functional recovery and developed brain atrophy that was larger in size than that seen in control mice [180]. In rats, the RXR agonist bexarotene attenuated neuroinflammation and improved neurological deficits after subarachnoid hemorrhage [181]. These studies highlight the important and protective role of RXR in cerebrovascular function, which further raises up the interest of how brain RXR in the regulation cardiovascular function.

### Retinoic acid-related orphan receptor (ROR)

The RAR-related orphan receptor (ROR) belongs to a subfamily of the THR, which is a subfamily of the NRs [182]. The ROR subfamily contains three members: RORα (NR1F1), RORβ, and RORγ.

RORα plays an essential role in the regulation of cardiovascular function including circadian rhythm, development, and metabolism [183–185]. One human retrospective study found that RORα mRNA expression levels in patients with acute myocardial infarction is significantly higher than that in patients with stable coronary artery disease [186]. Serum antibody levels of Claudin domain containing 1 (CLDND1)-derived peptides are elevated in patients with cerebral infection, CVDs or diabetes mellitus as compared to healthy controls. RORα overexpression in human brain endothelial cells enhanced CLDND1 transcript expression, while in contrast, siRNA-mediated knockdown of RORα significantly decreased CLDND1 transcription [187].

RORγt, has been shown to contribute to the development of hypertension in spontaneously hypertensive rat and may be potential therapeutic targets [188]. Mice with RORα deficiency demonstrated significantly augmented diastolic dysfunction and cardiac remodeling, which were associated with aggravated myocardial apoptosis, autophagy dysfunction, and oxidative stress, while restoration of cardiac RORα levels significantly improved cardiac functional and structural parameters [189]. Consistent with genetic manipulation, pharmacological activation of RORα by melatonin and SR1078 (a synthetic agonist) showed beneficial effects against diabetic cardiomyopathy, while the RORα inhibitor SR3335 significantly exacerbated cardiac impairments in diabetic mice [189]. Collectively, these findings suggest that cardiac-targeted manipulation of nuclear melatonin receptor RORα may hold promise for delaying diabetic cardiomyopathy development.

RORα is widely expressed in many tissues, including cerebellar Purkinje cells, the liver, thymus, skeletal muscle, skin, lung, adipose tissue, and kidney [190, 191]. RORγ has a similar broad pattern of expression but is observed at very high levels within the thymus. RORβ has a more restricted pattern of expression relative to the other RORs and is found in regions of the central nervous system (CNS) that are involved in the processing of sensory information, the retina and the pineal gland [192].

Previous studies reported that RORs in the circadian rhythm, metabolism, immune function, and tumorigenesis [185, 193, 194]. Mice with a loss-of-function mutation in RORα had alterations in the circadian oscillator, indicating an essential role for this receptor in normal circadian function [195]. Circadian misalignment causes adverse metabolic and cardiovascular consequences [196]. Current studies imply that brain RORs may play critical roles in the regulation of cardiovascular functions, directly or indirectly, but further studies are needed.

### Vitamin D receptor (VDR)

Vitamin D has a protective role in CVDs and vitamin D deficiency is associated with increased risk of hypertension, atherosclerosis, and heart failure [197]. It is noticed that a seasonality in patients suffering from CVDs could be attributed to the low 25-hydroxyvitamin D levels patients had in winter [198]. Vitamin D deficiency is associated with arterial stiffness, hypertension, left-ventricular hypertrophy, and endothelial dysfunction in patients with chronic kidney disease, as well as in normal subjects [199–201]. However, recent clinical intervention studies failed to prove the causal relationship between vitamin D supplementation and beneficial effects on cardiovascular health. In the RECORD (Randomised Evaluation of Calcium and/OR vitamin D) trial, daily vitamin D (800 IU) or calcium supplementation did not affect mortality, vascular disease, cancer mortality, or cancer incidence [202]. Another randomized, placebo-controlled trial found that inflammatory milieu was reduced in patients suffering from congestive heart failure, but left ventricular function remained unchanged after administration of 2000 IU vitamin D for nine months [203].

The vitamin D receptor (VDR), a member of the NR/steroid hormone receptor superfamily, functions as ligand-activated, transcriptional regulatory proteins. Thus, VDR selectively binds to 1,25-(OH)_2-_vitamin D_3_, the active form of vitamin D, and then forms a heterodimer with the RXR [204]. Previous immunohistochemical evidence showed that VDR was present in both neurons and glia of the human [205] and the rodent brains [206]. VDR has been noted in key dopaminergic brain regions in both the human brain [205] and the rat brain [206]. One study further showed that VDR is present in the nucleus of tyrosine hydroxylase-positive neurons in both the human and rat substantia nigra [207]. Emerging evidence indicates that brain VDR is critical in the vulnerability to central nervous system diseases including Parkinson’s Disease [208] and multiple sclerosis [209]. However, whether brain VDR regulates cardiovascular function is unclear.

### REV-ERB

The NRs REV-ERBα and -β regulate circadian rhythm and modulate glucose and lipid metabolism and inflammatory response [210–212]. REV-ERBs lack C-terminal helix, the canonical NR activation domain, and thus function as a transcriptional repressor [213].

A great number of studies highlight the importance of REV-ERB in circadian rhythm and metabolic functions including glucose homeostasis and energy homeostasis [212, 214–218], while emerging studies point out the important role of REV-ERBα/β in cardiovascular function [219–221]. Mice with cardiac-specific deletion of REV-ERBα/β and inducible ablation of REV-ERBα/β in adult hearts both displayed progressive dilated cardiomyopathy and lethal heart failure [219]. This study further identified that circadian disruption, which was implicated in heart diseases, was seen in the knockout mice [219]. Moreover, altered temporal patterns of cardiac REV-ERB gene expression was associated with the cardiac dilation severity in human hearts with dilated cardiomyopathy [219]. RER-ERB agonists, SR9009 and SR9011, were shown to improve hyperglycemia, dyslipidemia, and skeletal muscle oxidative capacity through modulation of mitochondrial number and oxidative function [211]. Recently, long-term treatment with SR9009 was reported to reduce atherosclerotic plaque in low-density lipoprotein receptor-deficient mice fed a Western diet [220]. Moreover, REV-ERB agonist SR9009 treatment significantly reduced post-myocardial infarction mortality and improved cardiac function through modulating inflammation and remodeling process [221]. These findings highlight the critical role of REV-ERBα/β in CVDs, especially the progression of myocardial infarction and heart failure.

REV-ERBα mRNA and protein were found in different brain regions including hippocampus, cortex, cerebellum, nigra, striatum, and hypothalamus in mice [222]. Accumulating evidence implies that the circadian NR REV-ERBα is involved in mood regulation. Altered midbrain REV-ERBα induces mania-like behavior and a hyperdopaminergic state and defective hippocampal function and memory [223, 224]. Most recently, the specific deletion of both REV-ERBs in the tuberal hypothalamic nuclei (including the ARC, VMH, and DMH) revealed the crucial role of REV-ERB in hypothalamic control of food intake and diurnal leptin sensitivity in diet-induced obesity [225]. Further studies are needed to explore whether central REV-ERB directly regulate cardiovascular function.

### Farnesoid X receptor (FXR)

The Farnesoid X receptor (FXR), also known as bile acid receptor, is a NR activated by bile acids. FXR regulates bile acid synthesis, conjugation, and transport, as well as various aspects of lipid and glucose metabolism [226]. Recent evidence shows that FXR is expressed in cardiomyocytes and endothelial cells and acts as a novel functional receptor in cardiac tissue, regulates apoptosis in cardiomyocytes, and contributes to myocardial ischemia/reperfusion injury [227]. Pharmacological inhibition or genetic ablation of FXR significantly reduced myocardial apoptosis, decreased infarct size, and improved cardiac function in ischemic/reperfused myocardium [227].

FXR expression was found in mouse and human brain [228, 229]. Further, multiple bile acids could be detected in the central nervous system [229–231], which may regulate FXR function in the brain. Recent studies have shown that FXR expressed in frontal cortex and hippocampus of mouse brain is involved in the pathogenesis of some neurological disorders, such as depression and Alzheimer's disease [230, 232, 233]. Besides, hippocampal FXR may help improving the insulin sensitivity in the Alzheimer's disease model rats, while FXR in the dorsal vagal complex (DVC) of the brain is involved in the insulin resistance in rats [230, 234]. It was shown that short-term HFD induces taurochenodeoxycholic acid (TCDCA, a FXR ligand) in the plasma and subsequently in the DVC of the brain induces insulin resistance in rats, while genetic knockdown or chemical inhibition of FXR in the DVC of HFD rats reversed insulin resistance [234]. More importantly, hippocampal FXR was found to regulate brain derived neurotrophic factor (BDNF) biosynthesis, which suggests that central FXR may play an important role in the regulation of metabolism considering the critical role of BDNF in the energy homeostasis [233, 235]. However, there is yet direct evidence to show that central FXR regulates cardiovascular function.

### Liver X receptor (LXR)

The liver X receptors α and β (LXRα and LXR β) are members of the NR family of proteins that play a crucial role in the regulation of cholesterol and lipid homeostasis [236, 237]. In the nucleus, LXRs form obligate heterodimers with the RXR and are bound to LXR response elements in regulatory regions of target genes. LXRα and LXR β are expressed in an overlapping but nonidentical pattern. LXRα is abundantly expressed in the liver, adipose tissue, heart, skeletal muscle, kidney, and lung while LXRβ is ubiquitously expressed [238]. The endogenous ligands for the LXRs are oxysterols, the oxidized derivatives of cholesterol. LXRs function as whole body cholesterol sensors [238].

Besides their established role in metabolic homeostasis and disease, there is mounting evidence suggests that LXRs have beneficial effects in the heart. The emerging role of LXRs in cardiac pathophysiology and heart failure was previously summarized [239]. LXRs prevents pathogenic accumulation of cholesterol in macrophages by enhancing the rate of cholesterol efflux [240]. Thus, LXRs exerts protective effects in the development of atherosclerosis and subsequent myocardial infarction and systolic heart failure. LXR agonist significantly reduces atherosclerosis in mouse models [241]. On the other hand, LXRs prevent against the presence of co-morbidities such as hypertension, atrial fibrillation, and diabetes [239]. These observations support the notion that LXRs may represent a novel therapeutic target for the treatment of human CVDs.

Both LXR isoforms are expressed in the brain [135] and play neuroprotective roles in the CNS. LXRs have been widely involved in the development of CNS and implicated in the regulation of brain function, such as anxiety [242] and neurodegenerative diseases [237, 243]. More importantly, LXRs are expressed in the hypothalamus, which suggested that LXR may exert metabolic regulation in the brain [244]. Recent studies found the knock-down LXRs in vivo or in LXRαβ^−/−^ mice activated TSH-releasing hormone (TRH) signaling in the PVH increased the activity of the hypothalamic-pituitary-thyroid axis, ultimately leading to the enhanced browning of subcutaneous adipose tissue thereby ameliorating obesity outcomes [245, 246]. LXRs also show neuroprotective roles in different experimental stroke models: stabilizing the integrity of brain capillaries, improving stroke outcome, and suppressing neuro-inflammation; and LXR activation protects hippocampal microvasculature through improving the microvascular architecture in Alzheimer Disease models [247–250]. These studies suggest the potentially preventative role of central LXR in the regulation of cardiovascular function.

### Small heterodimer partner (SHP)

Like DAX1, small heterodimer partner (SHP) is also an atypical orphan NR and acts as a negative regulator of receptor-dependent signaling pathways [251].

SHP is highly expressed in liver, heart, brain, pancreas, and small intestine [252]. In vitro study found that SHP upregulation upon high-fat feeding leads to lipid accumulation, insulin resistance and inflammation in cardiomyocytes [253]. In vivo study showed that SHP-null mice showed a cardiac hypertrophy. In addition, the metformin-induced antihypertrophic effect was diminished in SHP-null mice or by SHP small interfering RNA in cardiomyocytes [254]. SHP gene is regulated by several NRs, including FXR, liver receptor homolog-1, ERRγ, ERα, LXR, and SF-1 [252, 255, 256]. SHP itself is also an important modulator of nuclear signaling pathways by acting as a repressor antagonizing the activities of many NRs. Like DAX-1, SHP inhibits interaction with ER and its function [257, 258]. SHP mediates the anti-inflammatory actions of LXRs through differential regulation of receptor SUMOylation specifically in astrocytes, thereby revealing potential avenues for therapeutic development in diseases associated with brain inflammation [259]. Other functions in the CNS have not been reported yet.

### Constituitive androstane receptor (CAR)

The constitutive androstane receptor (CAR) is defined as xenobiotic NR that expressed in hepatocytes and activates genes involved in drug disposition, lipid homeostasis, and cell proliferation [260–262]. The metabolic function of CAR has been studied, which has a therapeutic potential in the prevention and treatment of type 2 diabetes and obesity [263, 264]. CAR was reported to interact with hypoxia inductile factor to regulate the hypoxia signaling for the development of CVDs [265]. Recent human [266, 267] and animal [268, 269] studies reported that mRNA and protein expression of CAR in the brain, including the cerebral cortex, hippocampus, amygdala, hypothalamus, and the basal ganglia. However, the role of brain CAR in the regulation of cardiovascular function is unknown.

### Steroidogenic factor 1 (SF-1)

Steroidogenic factor-1 (SF-1, Ad4BP) is a pivotal determinant of endocrine function within the hypothalamic-pituitary–gonadal axis and adrenal cortex and an essential factor in sex differentiation [270]. SF-1 transcripts were originally detected in adrenocortical cells, testicular Leydig cells, and ovarian theca and granulosa cells, and recently found in the dorsomedial part of ventromedial hypothalamic nucleus (VMH) neurons [39, 271, 272]. These findings raised the intriguing possibility that SF-1 playes critical roles that extend beyond the maintenance of steroidogenic capacity within the primary steroidogenic tissues.

Following studies proved this possibility. Embryonic deletion of SF-1 results in perinatal lethality and malformation of the VMH, suggesting that SF-1 is essential for the normal development of the VMH [271, 273, 274]. The VMH has been demonstrated to modulate the sympathetic nervous system (SNS) activity in the periphery. Rats with lesions in the VMH showed reduced sympathetic outflow and heart rate [275]. In contrast, VMH activation by electronic stimulation or drug injection could increase the SNS activity [276, 277]. One study showed that activation of the VMH, using the chemogenetic tool, the Designer Receptors Exclusively Activated Designer Drugs (DREADDs), exacerbated cardiac remodeling and increased systolic blood pressure in hypertension rats via increased the sympathetic nervous system activity [278]. VMH is an important nucleus in responding to emotional stress, which is also a risk factor for CVDs [279]. SF-1 neurons in VMH play a pivotal role in the regulation of glucose and energy homeostasis [39, 280]. But currently there are no studies to examine the role of SF-1 neurons in VMH in the regulation of cardiovascular function.

Many studies have shown that the deletion of various key factors specifically in the SF-1 neurons, such as leptin receptor, ERα, phosphoinositide 3-kinase and AMPK, etc. involved in the regulation of energy homeostasis and obesity [272, 281–286]. VMH may be the origin of leptin-mediated sympathoexcitation which contributes to obesity-related hypertension [287], but no cardiovascular effects were reported in these mutant mouse models.

### Liver receptor homolog‐1 (LRH‐1)

The orphan NR liver receptor homolog‐1 (LRH‐1) is highly expressed in liver, pancreas, and ovary [288]. Recent evidence further shows nuclear LRH-1 immunoreactivity in the heart, brown adipose tissue, and also ARH and PVH of the brain [289].

LRH-1 is constitutively active, which indicating its function occurs via interactions with co-activators and corepressors, as well as through posttranslational modifications, phosphorylation, and sumoylation [290]. Co-activators include PGC-1α, CBP, FXR, MBF-1, SRC-1/3, and phospholipids, while co-repressors include Prox1, SMRT, SHP, DAX1 [290]. FXR and SHP are NRs as discussed above.

A growing number of studies showing that LRH-1 plays critical roles in the regulation of development, cholesterol transport, bile acid homeostasis, and steroidogenesis [291], which are associated with cardiovascular functions. It has been shown that in vitro ligand of LHR-1, dilauroyl phosphatidylcholine, protects against hepatic steatosis and insulin resistance in mice fed with high-fat in an LRH-1–dependent manner [292]. Further study also suggested that LRH-1 is a critical component of the hepatic glucose-sensing system and integrates glucose and lipid homeostasis in the postprandial phase [293]. Recently, researchers also found that eliminating LRH-1 in the hepatocyte of the adult mice can profoundly affect the hepatic lipid metabolism and the glucose tolerance [294]. Heterozygous deletion of LRH-1 only causes mild body weight gain in HFD-fed mice [295]. In addition, using RNA interference and adenovirus-mediated overexpression, one study reported that LRH-1 directly regulates Apolipoprotein A1 (ApoA1) gene transcription [296]. ApoA1 is the primary protein component of high-density lipoprotein. Elevated ApoB and decreased ApoA1 are associated with increased risk of CVDs [297, 298]. Further studies will be needed to test whether LRH-1 directly regulates cardiovascular functions.

One recent study showed that LRH-1 is expressed in kisspeptin neurons in the arcuate nucleus but not in the anteroventral periventricular of the hypothalamus in female mice [299]. Kisspeptin is a neuropeptide, encoded by kisspeptin 1 (*Kiss1*) gene, which primarily acts as the regulator of reproductive functions [300]. Deletion of LRH-1 from kisspeptin neurons in mice led to decreased *Kiss1* expression in the arcuate nucleus, associated with decreased serum FSH levels, impaired follicle maturation, and prolongation of the estrous cycle [299]. It was recently reported that kisspeptin could change the morphology and structure of myocardial cells and regulate the expression of certain genes and proteins related to heart diseases [301]. LRH-1’s precise roles in the CNS and furthermore whether it directly regulates cardiovascular function remain to be elucidated.

## NRs without known cardiovascular functions

### Nerve growth factor 1B (NGF1B)-like receptors: Nur77, Nurr1, NOR-1

Nerve growth factor IB (NGF1B)-like receptors (nuclear hormone receptor 4A, NR4A) are a group of orphan NRs, comprising three members Nur77, Nurr1, NOR-1 [302, 303]. Since the endogenous ligand has not been identified, NGF1B-like receptors are considered to be constitutively active and the transcriptional activity is regulated mainly by the expression levels, and also by intracellular localization, modifications and protein–protein interactions [304].

All three members of the NR4A are expressed in CNS and peripheral tissues with high metabolic activity such as skeletal muscle, liver, heart and adipose tissues [302]. In the brain, the expression of NR4A in different brain area may show different functions, for example, in the hippocampus, NR4A contributes to memory formation [305], and in the hypothalamus, Nur77 mediates the anorexigenic effects of leptin [306]. Decreased expression of Nur77 in the hypothalamus has been identified in obese mice, which suggest that decreased hypothalamic Nur77 may contribute to development of obesity. Consistent with this, global Nur77 knockout mice develop obesity associated with increased food intake and decreased energy expenditure and are insensitive to the anorexigenic effects of leptin. More importantly, knockdown of Nur77 specifically in the hypothalamus blunts the actions of leptin on feeding and body weight [306]. Therefore, hypothalamic Nur77 may function as a positive modulator in leptin signaling and can contribute to the regulation of body weight balance. NOR-1 may also promote food intake and body weight gain through its actions in the brain, as the injection of a siRNA oligonucleotide against NOR-1 into the third cerebral ventricle markedly suppressed food intake and body weight in mice [307].

NR4A also regulates the dopamine neurons activity in the midbrain. Activation of the SNS leads to the release of its effectors, the catecholamines (Norepinephrine and epinephrine), from post-ganglionic cardiac sympathetic neurons. Norepinephrine and epinephrine are synthesized from the tyrosine and dopamine, in which tyrosine hydroxylase (TH) is the rate-limiting enzyme. In the brain of Nur77-knockout mice, TH mRNA expression and levels of the dopamine precursor are higher than WT mice, suggesting the enhanced TH activity [308]. Nurr1 is also essential for the development of midbrain dopamine neurons. Nurr1-knockout mice die soon after birth due to complete lack of midbrain dopaminergic neurons, and Nurr1 heterozygous mice have decreased TH expression and catecholamine levels in the midbrain [309, 310]. Therefore, Nurr1 is essential for both survival and final differentiation of dopaminergic neurons.

However, whether the deletion of NR4A in these neurons will induce any cardiovascular phenotype still need further investigation. Most recently, it is reported that the old Nurr1 knockout mice (both male and female) have higher heart rate without alteration in systolic blood pressure; young female Nurr1 knockout mice showed an increased heart rate in comparison to their WT littermates [311], but the molecular mechanism is still unclear.

### The germ cell nuclear factor (GCNF)

The orphan NR germ cell nuclear factor (GCNF), also known as RTR (retinoid receptor-related testis-associated receptor), is essential for embryonic development and reproduction [312–315]. In the mouse, GCNF is expressed in the developing nervous system, placenta, embryonic gonads, and adult ovaries and testes [316]. In vitro study established and characterized a *GCNF*^*−/−*^ embryonic stem cell line and reported that GCNF plays a central role in repressing the embryonic stem cell phenotype during RA-induced differentiation [317]. Loss of GCNF in the oocyte of female mice caused impaired fertility, which was due to prolonged diestrus of estrus cycle and aberrant steroidogenesis [315]. However, GCNF’s function in the development of obesity and CVDs is unknown.

### Testicular receptor (TR)

There are two forms of the testicular receptor (TR), TR2 and TR4. TR2 is robustly expressed in the developing olfactory epithelium and in more caudal regions of the brain, including the cortex, ventral forebrain structures, and thalamus [318]. TR4 is implicated in multiple pathological processes, including cerebellar development [319, 320], reproductive function [321, 322], and cancer development [323, 324]. Currently, there are no studies of TRs in the regulation of cardiovascular function.

### Pregnane X receptor (PXR)

Pregnane X receptor (PXR) is an important component of the body’s adaptive defense mechanism against toxic substances including foreign chemicals (xenobiotics) [325]. PXR is activated by many endogenous and exogenous chemicals including steroids, antibiotics, antimycotics, bile acids, hyperforin, and other compounds such as meclizine, cafestol and forskolin [326, 327]. One recent study reported that Bisphenol A significantly increased atherosclerotic lesion area in the aortic root and brachiocephalic artery in PXR-Humanized ApoE deficient mice [328]. This study may suggest the role of PXR in the development of atherosclerosis, but currently there are no studies regarding the brain PXR in cardiovascular control.

### Chicken ovalbumin upstream promoter transcription factor (COUP-TF)

Chicken ovalbumin upstream promoter transcription factors (COUP-TFs) are essential for the development of multiple tissues and organs, such as COUP-TFII is required for angiogenesis and heart development and COUP-TFI functions in neural development [329, 330]. COUP-TFII is a NR classified as an orphan due to the lack of a known natural ligand. COUP-TFII affects mitochondrial function, impairs metabolic remodeling, and has a key role in dilated cardiomyopathy [331]. Increased COUP-TFII expression is associated to brain arteriovenous malformations in humans [331]. COUP-TFII is important for migration of cortical interneurons from caudal ganglionic eminence (CGE) to the neocortex in rodents and human fetal brain [332, 333]. By using focal in utero labeling of the preoptic area (POA), one study [334] showed that switching on/off the transcription factor COUP-TFII and the receptor Neuropilin-2 (Nrp2) directs the POA-derived neurons toward either the amygdala or cortex. This study revealed an essential role of COUP-TFII/Nrp2 expression dynamics in the development of the amygdala and cortex. However, how brain COUP-TFII regulates cardiovascular function is unknown.

### DAX-1

DAX-1 [dosage-sensitive sex reversal-adrenal hypoplasia congenita critical region on the X chromosome, gene 1] is an atypical orphan NR. DAX1 is expressed in the adrenal gland, gonad, pituitary gland, and hypothalamus [335].

DAX-1 emerges as a global negative regulator of genes involved in steroid hormone production and metabolism in steroidogenic tissues [336–340]. Previous study in *Ahch* (the mouse DAX-1 homologue) knockout mice indicates that DAX-1 may play a direct role in spermatogenesis [341]. Recent results obtained in patients of adrenal hypoplasia congenita suggest that DAX-1 is also required for spermatogenesis in humans, independent of gonadotropin and testosterone production [342]. Impaired spermatogenesis is a risk factor for CVDs [343]. It has been reported that DAX-1 can inhibit SF-1 transcriptional activity [337, 338]. In addition, DAX1 has been shown to repress the transcriptional transactivation of ER [339], AR [344] and PR [340]. Considering that all these NRs, especially ER, AR and PR, play key roles in the regulation of cardiovascular functions as described previously, DAX-1 may also be involved in this process which is unknown yet.

### Tailless (TLX)

The orphan NR tailless (TLX) is exclusively expressed in the brain [345]. TLX has been identified as a fundamental regulator of adult neural stem cells and neurogenesis which involved in the learning and behavior [346, 347]. It has been reported that the protein level of TLX in the striatum, cortex, and hippocampus of mice was attenuated when fed with HFD [348]. However, the function of TLX in the development of obesity and CVD is not clear.

### The photoreceptor cell-specific nuclear receptor (PNR)

The photoreceptor cell-specific NR (PNR) is a member of the nuclear receptor super family of intracellular transcription factors. PNR is exclusively expressed in the retina [349] and plays a role in the regulation of signaling pathways intrinsic to the photoreceptor cell function [349]. There is no report about effects of PNR on obesity and CVD.

### Hepatocyte nuclear Factor‐4 (HNF4)

Hepatocyte nuclear factor‐4 (HNF4α) is a highly conserved member of the NR superfamily. HNF4α is expressed in liver, kidney, small intestine, colon, and pancreas [350, 351]. The essential fatty acid linoleic acid has been identified as the endogenous ligand for HNF4 [352]. Like LXR, HNF-4 emerged recently as another key regulators in reverse cholesterol transport, which prevents lipid accumulation and represents a promising target of new therapeutic agents for the prevention and treatment of metabolic diseases and atherosclerosis [353]. However, the studies in HNF-4 are much less than LXR and its central role is not clear.

## Summary

Given the great number of NRs, the abundant expression of NRs expressed in many brain regions and peripheral tissues, and their broad roles in the regulation of cardiovascular functions, here we summarized the current pharmaceutical and genetic evidence, brain expression, functional brain regions and effects on cardiovascular functions in Table 1. Numerous studies demonstrated the abundant expression of the NRs in almost all brain regions including cerebral cortex, cerebellum, hypothalamus, thalamus, pituitary gland, pineal gland, amygdala, hippocampus, and midbrain [7–9, 14, 39, 53–55, 104, 134, 135, 205–207, 271, 272, 289, 305, 306, 318]. However, the functional studies from the perspective of brain NRs in the regulation of cardiovascular functions are only available for a few NRs [12–14, 58–62, 88]. Briefly, in this review we encapsulated that the ER in RVLM and PVH decreasing SAP and protecting against hypertension, the MR in NTS and projection neurons increasing salt appetite and satiation, and the THR in parvalbuminergic neurons of anterior hypothalamus augmenting heart rate and blood pressure in Fig. 1. In consideration of the many other NRs with unknown brain connections but have clear cardiovascular regulations, we call more efforts in this field. One of the reasons for these efforts is that NRs could be suitable therapeutic targets for CVDs. Raloxifene, a selective estrogen receptor modulator (SERM) was reported to favorably reduce serum levels of low-density lipoprotein (LDL) cholesterol in postmenopausal women in a randomized, placebo-controlled clinical trial [354]. One contemporary primary prevention cohort study showed that elevated LDL cholesterol had the highest absolute risk of CVDs including myocardial infarction and atherosclerosis [355]. Moreover, pravastatin, a medication used to lower cholesterol, was found to reduce the incidence of fatal coronary event [356]. Besides SERM, PPARγ agonist is another example to imply the potential of NRs in treatment of CVDs. Clinical trials have found that PPARγ agonists pioglitazone and rosiglitazone could reduce systolic and/or diastolic blood pressure, respectively [357, 358]. Long-term reduction of blood pressure might be expected to decrease the risk of congestive heart failure [359]. These studies underscored the importance of NRs in the treatment of CVDs and the necessity to improve our understanding of the mechanisms of NRs in the cardiovascular control, especially via the brain connection. Overall, a better understanding of the cardiovascular function of brain NRs may facilitate the development of novel targeted therapies in future.

**Table 1:** Summary of brain NR effects on cardiovascular function and underlying mechanisms

NR	Pharmaceutical study	Genetic study	Brain expression	Functional brain regions	Effects on CV function
*I. Brain NRs that regulate cardiovascular functions*					
ER	Yes	Yes	The olfactory bulb, cerebral cortex, septum, preoptic area, bed nucleus of the stria terminalis, amygdala, paraventricular hypothalamic nucleus, thalamus, ventral tegmental area, substantia nigra, dorsal raphe, locus coeruleus, and cerebellum	1. The rostral ventrolateral medulla (RVLM) 2. The paraventricular nucleus of the hypothalamus (PVH)	1. ERβ, instead of ERα, induced significant decrease in systemic arterial pressure (SAP) and the power density of the vasomotor components of SAP signals 2. Both PVH and RVLM ERβ, instead of ERα in these nuclei, contribute to the protective effects of estrogen against aldosterone-induced hypertension
MR	Yes	Yes	The dorsal and ventral hippocampal CA1-4 pyramidal cell fields and the dentate gyrus, amygdala, and lateral septum	NTS and projection neurons	MR-NTS regulates salt appetite and projection neurons including parabrachial nucleus, amygdala, paraventricular nucleus, hippocampus, and the bed nucleus of the striae terminalis (BNST) signal salt satiation
THR	No	Yes	In mice, THRα1 mRNA is predominantly expressed in the heart and brain, whereas THRβ1 is expressed in peripheral tissue	Parvalbuminergic neurons in the anterior hypothalamus	THR in parvalbuminergic neurons is required for proper heart rate and blood pressure
*II. NRs that regulate cardiovascular functions with unclear brain connection*					
ERR	Yes	Yes	ERRα and ERRγ are both widely expressed in the brain, whereas ERRβ expression is restricted to the hindbrain	Unclear	ERRα and γ in heart after birth are required for normal cardiovascular function; ERRβ is involved in maintaining maximal ATP generation in contracting cardiomyocytes
AR	No	Yes	The bed nucleus of the stria terminalis, lateral septum, medial amygdala, medial preoptic area, and ventromedial hypothalamic nucleus	Unclear	The androgen-AR system regulates normal cardiac growth, cardiac adaptive hypertrophy, and fibrosis during the process of cardiac remodeling under hypertrophic stress
GR	Yes	Yes	The hippocampus and NTS	Unclear	Glucocorticoid administration increases blood pressure and contributes to the exacerbation of a cluster of cardiovascular risk factors
PPAR	Yes	Yes	PPARα and PPARβ/δ mRNA and protein are found to express in prefrontal cortex, nucleus accumbens, amygdala and ventral tegmental area; PPARγ immunoreactivity was primarily observed in hypothalamic arcuate and ventromedial neurons and was also present in the hypothalamic paraventricular nucleus, the lateral hypothalamic area, and tyrosine hydroxylase-containing neurons in the ventral tegmental area	Unclear	Like PPARα, PPARβ/δ improves cardiac function and ameliorate the pathological progression of cardiac hypertrophy, heart failure, cardiac oxidative damage, ischemia–reperfusion injury, lipotoxic cardiac dysfunction and lipid-induced cardiac inflammation
PR	Yes	Yes	Hypothalamic arcuate nucleus and anteroventral periventricular nucleus, hippocampus, and cortex	Unclear	Progesterone exhibits beneficial effects on the cardiovascular function such as lowers blood pressure, inhibits coronary hyperactivity, and has powerful vasodilatory and natriuretic effects
RAR	Yes	Yes	RARα is found in cortex and hippocampus and RARβ and RXRγ are both highly expressed in the dopamine-innervated areas caudate/putamen, nucleus accumbens and olfactory tubercle	Unclear	RA carries out essential roles in heart development and after birth in the heart’s remodeling response to injury and disease
RXR	No	Yes	Like above	Unclear	RAR/RXR regulates cardiovascular function including heart development, normal conductive system, and cardiac function
ROR	Yes	Yes	RORα and γ are found in cerebellar cortex and other peripheral tissues while RORβ is found in pineal body	Unclear	RORs are critical in in the regulation of cardiovascular function including myocardial infarction, cardiac remodeling, etc
VDR	Yes	Yes	The nucleus of tyrosine hydroxylase-positive neurons in both the human and rat substantia nigra	Unclear	Vitamin D deficiency in humans is associated with arterial stiffness, hypertension, left-ventricular hypertrophy, and endothelial dysfunction in patients with chronic kidney disease, as well as in normal subjects
REV-ERB	Yes	Yes	Hippocampus, cortex, cerebellum, nigra, striatum, and hypothalamus	Unclear	REV-ERBα/β controls the progression of myocardial infarction and heart failure
FXR	No	Yes	Frontal cortex and hippocampus	Unclear	FXR is expressed in cardiomyocytes and endothelial cells and acts as a novel functional receptor in cardiac tissue, regulates apoptosis in cardiomyocytes, and contributes to myocardial ischemia/reperfusion injury
LXR	Yes	Yes	Hypothalamus, PVH	Unclear	LXRs exerts protective effects in the development of atherosclerosis and subsequent myocardial infarction and systolic heart failure
SHP	No	Yes	Unclear	Unclear	SHP is required for normal cardiovascular function
CAR	No	No	The cerebral cortex, hippocampus, amygdala, hypothalamus, and the basal ganglia	Unclear	CAR interacts with hypoxia inductile factor to regulate the hypoxia signaling for the development of cardiovascular disease
SF-1	Yes	Yes	Ventromedial hypothalamic nucleus (VMH)	VMH	Lesions in the VMH showed reduced sympathetic outflow and heart rate; VMH activation by electronic stimulation or drug injection could increase the SNS activity
LRH-1	No	Yes	Kisspeptin neurons in the arcuate nucleus	Unclear	LRH-1 directly regulates ApoA1 and decreased ApoA1 is associated with increased cardiovascular disease
*III. NRs without known cardiovascular functions*					
NGF1B	Yes	Yes	Hypothalamus and hippocampus	Unclear	Unclear
GCNF	No	Yes	The developing nervous system, placenta, embryonic gonads, and adult ovaries and testes	Unclear	Unclear
TR	No	No	the developing olfactory epithelium and in more caudal regions of the brain, including the cortex, ventral forebrain structures, and thalamus	Unclear	Unclear
PXR	No	Yes	Unclear	Unclear	Unclear
COUP-TF	No	No	POA-derived neurons	Unclear	Unclear
DAX-1	No	No	Hypothalamus	Unclear	Unclear
TLX	No	No	Striatum, cortex, and hippocampus	Unclear	Unclear
PNR	No	No	Unclear	Unclear	Unclear
HNF4	No	No	Unclear	Unclear	Unclear

The emerging role of central NRs in the regulation of cardiovascular functions have gradually drawn our attention. Some NRs show great potential to regulate brain–heart interactions, yet we must admit that we are in the very early phase to establish the causative role of brain NRs in the etiology of the CVDs. The function of NRs in the brain is highly dependent on the brain region, cell type, or developmental stages, thus future studies using animal models with temporally controlled genetic manipulations in a specific brain region, cell type, or a circuit-related neuron population would help clarify their functions.

## Data Availability

Not applicable.
